# Mattering Mediates Between Fairness and Well-being

**DOI:** 10.3389/fpsyg.2021.744201

**Published:** 2021-11-03

**Authors:** Michael P. Scarpa, Salvatore Di Martino, Isaac Prilleltensky

**Affiliations:** ^1^School of Education and Human Development, University of Miami, Coral Gables, FL, United States; ^2^School of Social Sciences, University of Bradford, Bradford, United Kingdom

**Keywords:** mattering, fairness, well-being, social justice, dignity, SEM, multidimensional measure

## Abstract

Research has suggested a fundamental connection between fairness and well-being at the individual, relational, and societal levels. Mattering is a multidimensional construct consisting of feeling valued by, and adding value to, self and others. Prior studies have attempted to connect mattering to both fairness and a variety of well-being outcomes. Based on these findings, we hypothesize that mattering acts as a mediator between fairness and well-being. This hypothesis was tested through Covariance-Based Structural Equation Modeling (CB-SEM) using multidimensional measures of fairness, mattering, and well-being. Results from a Latent Path Analysis conducted on a representative sample of 1,051U.S. adults provide support to our hypothesis by revealing a strong direct predictive effect of mattering onto well-being and a strong indirect effect of fairness onto well-being through mattering. Results also show that mattering is likely to fully mediate the relationship between fairness and multiple domains of well-being, except in one case, namely, economic well-being. These findings illustrate the value of a focus on mattering to understand the relationship between fairness and well-being and to provide future directions for theory, research, and practice. Theoretical implications for the experience of citizenship and participation, along with cross-cultural considerations, are also discussed.

## Introduction

Despite decades of surging interest in well-being, there is still a need to understand the role that fairness and justice play in human flourishing (e.g., Greenberg and Colquitt, 2013; Prilleltensky, 2014; Yean, 2016; Di Martino and Prilleltensky, 2020). Although there is a robust literature on the psychology of social justice (Lind, 2020), especially in the context of work (Ybema and van den Bos, 2010), we still lack a full picture of how fairness impacts wellness. A promising new development is the emerging research on mattering (Schlossberg, 1989; Elliott et al., 2004; Flett, 2018; Prilleltensky, 2020; Prilleltensky and Prilleltensky, 2021). In particular, mattering has potential as a bridging concept that helps explain how fairness produces wellness at the individual, community, and societal levels. We develop in this paper the argument that mattering plays a mediating role between fairness and well-being.

### Well-being

Well-being (used interchangeably with wellness and flourishing here) is the subject of a vast and transdisciplinary literature (Arcidiacono and Di Martino, 2016). Within psychology, the study of well-being emerged in correction to the over-reliance on deficit models of mental health (Huppert, 2009). Over the years, well-being has earned a prominent place in disciplines such as positive, philosophical, child and family, social, community, and organizational psychology. It is frequently divided into hedonistic, or emotion-oriented; and eudaimonic, or meaning-oriented (Ryff and Singer, 1998).

Despite this variety, it is possible to identify key themes and features of the well-being landscape which can inform our discussion. First, well-being is about what is good for people (Crisp, 2001); it represents an ideal “positive state of affairs brought about by the simultaneous and balanced satisfaction of diverse objective and subjective needs of individuals, relationships, organizations, and communities” (Prilleltensky, 2012 p. 2). As this definition suggests, it can be thought of as having subjective (e.g., life satisfaction) and objective (e.g., life expectancy) elements (Diener and Suh, 1997; Oswald and Wu, 2010). Next, well-being is most often discussed in terms of multiple dimensions. These dimensions can be broad, as in Diener’s (1984) tripartite model (life satisfaction, positive affect, and negative affect) or highly specific, as in Ryff’s (1989) six-factor model of psychological well-being (autonomy, self-acceptance, positive relationships, environmental mastery, personal growth, and purpose in life). Finally, well-being is experienced by individuals in multiple domains of life (Prilleltensky et al., 2015) and can be understood as a function of social and ecological contexts as much as individual characteristics (Kelly, 2000; McGregor et al., 2003).

Well-being is fostered by the satisfaction of diverse needs in various areas of life (Deci and Ryan, 2011). These include physiological needs like sleep, psychological needs like autonomy (Sheldon and Gunz, 2009), relational needs like belonging (Baumeister and Leary, 2017), material needs like housing, and existential needs like purpose in life. This diversity underlies the I COPPE (i.e., Interpersonal, Community, Occupational, Physical, Psychological, and Economic well-being) model of well-being (Prilleltensky et al., 2015) used in the present study. This model, which is expressed in an assessment tool detailed below, understands subjective well-being as experienced in the overall, interpersonal, community, occupational, physical, psychological, and economic domains.

#### Cross-Cultural Considerations

Beyond these distinctions, substantial evidence has been furnished suggesting that well-being varies both conceptually and in its determinants across cultures (Oishi and Kurtz, 2011). For instance, researchers have suggested that some cultures may construct well-being from a more relational standpoint than others (Kitayama et al., 2010) and that collectivist cultures are more likely to ground well-being in experiences of social harmony than individualist societies (Kwan et al., 1997). Different cultures may also report varying levels of well-being due to factors including individualism or collectivism, the conceptualization of the self (Suh and Oishi, 2002), and cultural factors influencing item response (Kitayama et al., 2010; Cummins, 2019). However, the literature also provides evidence in support of basic construct equivalence and cross-cultural comparison at both the individual and societal levels (Boarini et al., 2014; Disabato et al., 2016; Aschauer, 2019).

As such, we view this and the other main constructs in our paper through what Lomas (2015) has termed a “universal relativism” lens. This approach favors a synthesis of universalist and relativist perspectives which allows room for deep similarities to be expressed differentially by culture. Under such a schema, basic determinants, such as working conditions, family relationships, and community, might contribute across cultures to well-being. However, the ways in which they do so might be mediated by culture-specific factors, such as values, norms, and tradition. Recent evidence taken from the Gallup World Poll (Joshanloo and Jovanović, 2021) appears to align with this perspective in the case of subjective well-being. As shall be discussed, we understand both mattering and fairness in similar terms.

### Mattering

Mattering is related to one’s experiences of feeling valued by, and adding value to, self and others (Prilleltensky, 2020). It can be considered a fundamental need (Flett, 2018) as well as part of the common good (Prilleltensky, 2020). Most contemporary psychological work on mattering can be traced to Rosenberg and McCullough’s articulation (Rosenberg and McCullough, 1981; Jung, 2015). Their conception portrayed mattering as an interpersonal construct composed of attention from others, importance to and dependence upon them, and, later, ego-extension and being missed. Numerous others have introduced further elaborations of mattering and extended their focus to different domains of life. For instance, Schlossberg (1989) expanded upon Rosenberg and McCullogh’s articulation by incorporating attention as a fifth dimension. Later, Elliott et al. (2004) demonstrated the empirical validity of a mattering measure based on this tradition, distilling the construct to three factors: awareness, importance, and reliance. Others have since expanded the assessment of mattering into the workplace (Jung and Heppner, 2017) and explored its relevance in contexts ranging from counseling relationships (Rayle, 2006) to social crises (Flett and Zangeneh, 2020).

More recently, authors have advocated an understanding of mattering which goes beyond feeling significant to others and incorporates the contributions that one can make (Jung, 2015; Prilleltensky and Prilleltensky, 2021; Reece et al., 2021). It has been increasingly recognized that mattering is important in various domains of life (i.e., personal, interpersonal, and occupational; Prilleltensky, 2020). Though most research has taken place in the context of interpersonal relationships, researchers have also demonstrated the importance of mattering in the workplace (Reece et al., 2021), in the community (Olcoń et al., 2017), and even to the self (Prilleltensky, 2020). Mattering has also been theorized as a contested construct related to social justice and the public good. For example, it has been put forward as the antithesis of social marginality (Schlossberg, 1989) and dispossession (Morrill and Tuck, 2016). Further, it has been suggested that the struggle to matter has great explanatory value for the study of right-wing populism, climate inaction, and opportunity hoarding (Prilleltensky, 2020). Hence, mattering has relevance beyond psychological dynamics and interpersonal relationships. Incorporating these insights, this paper will focus on Prilleltensky’s conceptualization of multidimensional mattering, defined as the synergistic balance of feeling valued and adding value across intrapersonal, relational, occupational, and community domains of life (Prilleltensky, 2020).

Hitherto, limited cross-cultural investigation into mattering has emerged (e.g., Demir et al., 2012; Taniguchi, 2015). Nevertheless, there is reason to expect that mattering experiences would differ between cultures. First, cross-cultural literature has demonstrated variability with respect to related constructs, including sense of community (Brodsky, 2009; Barbieri and Zani, 2015) and belonging (Chiu et al., 2016). Additionally, several empirical studies have demonstrated the existence of within-country demographic group differences in mattering (Scarpa et al., 2021a), some of which point to the role of cultural elements such as religiosity (Lewis and Taylor, 2009).

### Fairness

Fairness has been called “*the* most essential rule in social engagement,” (Sun, 2013 p.17) and conceptualized as justice in action (Prilleltensky, 2014). There is evidence that humans are fundamentally motivated to seek out and appreciate fairness (Montada, 2003; Brosnan and de Waal, 2014). In this paper, we focus on fairness as the application of distributive and procedural justice (Rawls, 1991; Ambrose and Arnaud, 2005; Lucas et al., 2011).

Procedural justice, as the name suggests, involves questions of fair process (Lind and Tyler, 1988), which occur whenever people are treated with respect and decisions are fairly and transparently made (Blader and Tyler, 2003). Distributive justice, on the other hand, is concerned with allotment of outcomes (Cohen, 1987). While distributive justice between individuals is certainly possible, it is more frequently invoked in discussions of macro-level social justice (e.g., Hülle et al., 2018). Meanwhile, procedural justice seems most frequently to be investigated in relational, legal, and workplace contexts (e.g., Greenberg and Tyler, 1987). Importantly, however, both have relevance across life domains (Prilleltensky, 2013). Although scholars have often engaged separately with these concepts, they can also be understood as complementary (Hauenstein et al., 2001; Ambrose and Arnaud, 2005; Lucas et al., 2011).

Beyond these formulations, researchers have suggested additional types of fairness, including relational, cultural, epistemic, and corrective justice. Fairness, like mattering, can be experienced by individuals across different domains of life (Duff, 2016). Indeed, fairness has been investigated in great detail between individuals (Bazerman et al., 1995), in the workplace (Bettencourt and Brown, 1997; Greenberg, 2011), in the home (Kawamura and Brown, 2010), and in society at large (Sadurski, 1985; Fondacaro and Weinberg, 2002).

Finally, fairness has been the subject of substantial cross-cultural analysis. We concur with Leung and Stephan (2001) in suggesting that justice can be understood as both universal and culture-specific. While justice is a universal motive, “culture may create drastic differences in what goes into the justice equation” (P. 400). Hence, the “universal relativism” perspective discussed above appears applicable to fairness as well. Among sources of difference, cultural values (Wang and Yao, 2011), power distance (Kim and Leung, 2007), and culturally influenced self-construal (Brockner et al., 2000) have all been shown to significantly influence fairness judgments.

### The Connection Between Mattering, Fairness, and Well-being

Despite cultural differences in their expression, each of the above constructs can be understood as a fundamental motivation, which is experienced by individuals across multiple domains of life. It is no surprise, then, that each has been connected theoretically and empirically to the others. In what follows, we briefly outline key connections between these constructs relevant to our hypothesized mediation model.

#### Fairness and Well-being

Theoretical arguments connecting wellness and fairness can be found in diverse literatures. Key to most are two notions: first, that humans have a fundamental need for fairness; second, that fairness helps stabilize beneficial social arrangements. In community psychology, it has been argued that justice helps produce well-being across ecological levels by promoting salutary conditions, improving relationships, and avoiding social comparison and status-based harm (Prilleltensky, 2012, 2013). Public policy authors have put forward that justice enhances well-being by strengthening democracy and faith in institutions (von Heimburg et al., 2021). Virtue ethics and existential psychology, meanwhile, suggest that justice helps advance flourishing by enhancing cooperation and upholding beneficial norms (Fowers et al., 2021).

This connection is also borne out by the evidence furnished by various studies. Among individuals, experiences of discrimination – a form of unfairness – have been linked to increased loneliness, depressive symptoms, and heart disease, among other negative outcomes (Mays et al., 2007; Williams et al., 2012; Priest et al., 2014). More general experiences of unfairness have been linked to reduced mental health functioning, increased depression, and drug use (Resnicow et al., 2021). In the workplace, unfair treatment has been connected to poor health and burnout (Daniels et al., 2017; Islam et al., 2021) as well as reduced employee satisfaction (Bettencourt and Brown, 1997; Lawson et al., 2009). Finally, researchers have introduced evidence that higher social justice index scores are correlated at the national level with higher life satisfaction (Di Martino and Prilleltensky, 2020). This finding builds upon literature connecting macro-level inequality to negative outcomes including worse mental health and increased violence (Subramanian and Kawachi, 2006; Wilkinson and Pickett, 2010; Rambotti, 2015).

#### Mattering and Well-being

The most important rationale for a connection between mattering and well-being lies in the basic necessity of mattering and its components. Feeling valued is comprised of such fundamental psychological and relational needs as belonging and secure attachment, while adding value is related to autonomy, self-determination, and self-efficacy (Prilleltensky, 2020). A robust literature links each of these constructs to well-being (e.g., Reis et al., 2000).

Beyond theory, a growing body of empirical evidence demonstrates the importance of mattering to well-being across the lifespan and in various life domains. For young children, attachment to parents is a basic relational need whose fulfillment is reflected in and clarified by mattering (Charles and Alexander, 2014; Flett et al., 2020; Prilleltensky, 2020). For adolescents, mattering to the community helps protect against suicidal ideation and behavior while increasing physical exercise (Murphey et al., 2004; Olcoń et al., 2017). Among university students, mattering creates belonging and alleviates marginalization (Schlossberg, 1989; Huerta and Fishman, 2014). For adults, mattering inspires connection with others (Zeeb and Joffe, 2020) and improves workplace engagement and job success, while reducing burnout (Flett and Zangeneh, 2020; Reece et al., 2021). Mattering also improves the transition to retirement communities (Froidevaux et al., 2016) and protects one’s health in later life by moderating the relationship between allostatic load and age (Taylor et al., 2019).

More generally, mattering has been identified as a buffer against academic stress (Rayle and Chung, 2007) and stress in general (Turner et al., 2004); a broad correlate of physical and mental health (Flett, 2018); a protective factor during life transitions (Schlossberg, 1989; Froidevaux et al., 2016); a predictor of job satisfaction and intent to leave (Reece et al., 2021); a buffer against suicidal ideation and behaviors (Elliott et al., 2004; Murphey et al., 2004); a protective factor against internalized gay ageism (Wight et al., 2015); and a contributor to persistence and belonging on college campuses (Palmer and Maramba, 2012; Huerta and Fishman, 2014). In short, the relationship between mattering and well-being is wide-ranging and well-documented.

While most research has occurred in Western, English-speaking populations, connections between mattering and well-being have also been demonstrated in Turkish (Demir et al., 2012), Malaysian (Kam and Prihadi, 2021) and Japanese samples (Taniguchi, 2015), and among Spanish-speaking respondents in the United States (Dueñas and Gloria, 2017, 2020; Huerta and Fishman, 2014). In addition, evidence has suggested that religiosity contributed more to mattering in African American than in White respondents in a U.S. sample (Lewis and Taylor, 2009). Hence, while there is reason to believe the association between mattering and well-being is broadly shared, reasons for this association may vary between groups.

#### Mattering and Fairness

So far, few empirical studies have investigated connections between mattering and fairness or justice (e.g., Kawamura and Brown, 2010; Lachance-Grzela, 2012). Conceptually, however, there is ample reason to expect a relationship. This can be seen most clearly by reviewing several concepts, such as dignity, self-determination, and belonging, which have been connected to both constructs.

Dignity is the notion that people are inherently entitled to respectful treatment. In our terms, it is the requirement and practice of honoring the mattering of self and others. Nussbaum’s insight that securing dignity requires a capabilities-based approach to justice – which insists on what people “are actually able to do and to be” (Nussbaum, 2000 p. 5) – suggests that dignity requires the ability to add value. Meanwhile, within psychology, Hicks has positioned fairness as an “essential element” of dignity (Hicks, 2011). This perspective is echoed by philosopher Michael Sandel, who writes, “justice requires itself to uphold the human rights of all people … simply because they are human beings.” (Sandel, 2010 p. 123). Dignity has also been put forth as a key aspect of procedural justice (Byers, 2016), a critical aspect of justice in general (Honneth, 2001), and the basis of human rights (Fraser, 2010). In other words, fairness ensures dignity, which in turn contributes to mattering.

Related to dignity is self-determination, a value which supports feeling valued and adding value. Self-determination theory (SDT) links satisfaction of individual needs, such as autonomy, relationship quality, and competence to overall well-being (Sheldon et al., 2004; Deci and Ryan, 2011). Evidence suggests that procedural justice judgments are influenced by the satisfaction of autonomy needs (van Prooijen, 2009). Furthermore, fairness has been shown to communicate inclusion and interact with perceived social status (Tyler and Blader, 2002; van Prooijen et al., 2004). Beyond psychology, a longer history in contemporary social philosophy (e.g., Young, 1979) considers self-determination of both individuals and communities to be foundational to justice. A collective understanding of self-determination predicates adding value on conditions of fairness (Murphy, 2014). Hence, a clear link between fairness, mattering, and well-being at both individual and collective levels passes through self-determination.

Another important connection is between mattering and belonging. Fromm (1994) suggests that we have a desire to belong in order to avoid a sense of insignificance (*cf*. Zeeb and Joffe, 2020). Dueñas and Gloria (2017) echo this idea in designating belonging a social dimension of mattering. Meanwhile, researchers have connected greater procedural fairness to an increased sense of group identification, need to belong, and inclusion (MacCoun, 2005). Most recently, Valcke et al. (2020) conducted two experimental studies with racialized minority participants whose outcomes suggest that procedural fairness produces belonging by enhancing trust and feelings of being accepted. It seems possible, then, that fairness reassures us that we matter by demonstrating that we belong and by helping our communities cohere.

## Purpose and Aims

Mattering, fairness, and well-being are core human motivations. Each is best understood in multidimensional terms across domains of life. Further, each can be bridged with each of the others conceptually and empirically. The literature suggests that fairness bolsters mattering, which, in turn, is crucial for well-being. Hence, there seems to be value in empirically investigating the relationships among fairness, mattering, and well-being. Therefore, the purpose of the study was to examine the relationships among fairness, mattering, and well-being in the adult population of the United States of America.

### Hypotheses

Given the depth of established connections between mattering, fairness, and well-being and in view of the strong theoretical rationale connecting fairness to mattering outlined above, we hypothesize that mattering mediates the relationship between fairness and well-being. However, given the rich diversity of findings connecting fairness and well-being, we also expected a direct connection between the two constructs. Therefore, our study will test the following hypothesis:

H1: Multidimensional mattering, as measured by the Mattering in Domains of Life Scale (MIDLS) fully mediates the relationship between multidimensional fairness, as measured by the Multidimensional Fairness Scale (MFS) and multidimensional well-being, as assessed by the I COPPE scale short form.

## Materials and Methods

Approval for this study was obtained under University of Miami Institutional Review Board ID 20200295. All procedures performed in this study were in accordance with the approved protocol, ethical standards of the institution, and the 1964 Helsinki declaration, subsequent amendments, and comparable ethical standards.

### Participants and Recruitment

Recruitment was conducted online in partnership with Qualtrics, an online survey administration and panel recruitment company. The researchers contracted with Qualtrics, who monitored survey responses and enforced demographic quotas to obtain a representative U.S. sample. Surveys were distributed by administration companies partnered with Qualtrics to respondents who volunteered to take online surveys. Sampling was stratified by each of seven demographic variables, outlined below under Instruments and in Table 1, and quotas for each answer option were employed to obtain a representative U.S. sample.

**Table 1:** Participant demographics.

Variable		*n*	%
**Age**			
	18–25	124	11.9
	26–34	188	18.0
	35–54	365	34.9
	55–64	168	16.1
	65 or over	200	19.1
**Gender**			
	Female	520	49.8
	Male	523	50.0
	Other	2	0.2
**Annual Household Income**			
	$0–$24,999	207	19.8
	$25,000-49,999	267	25.6
	$50,000–$74,999	213	20.4
	$75,000–$99,999	139	13.3
	$100,000–$149,999	100	9.6
	$150,000–$199,999	53	5.1
	$200,000+	66	6.3
**Ethnicity**			
	White/Caucasian	679	65.0
	Hispanic/Latino(a)(x)	135	12.9
	Black/African American	136	13.0
	Asian	53	5.1
	Native American	21	2.0
	Pacific Islander	5	0.5
	Other	16	1.5
**Education level completed**			
	Grammar School	8	0.8
	High School or Equivalent	290	27.8
	Vocational/Technical School (2Year)	85	8.1
	Some College	219	21.0
	College Graduate (4year)	193	18.5
	Master’s Degree (MS)	85	7.9
	Doctoral Degree	16	1.5
	Professional Degree (MD, JD, etc.)	19	1.8
	Other	130	12.4
**What is your current marital status?**			
	Married	521	49.9
	Single	283	27.1
	Living With Partner	97	9.3
	Divorced	87	8.3
	Separated	20	1.9
	Widowed	37	3.5
**What is your employment status?**			
	Full Time	431	41.2
	Part Time	140	13.4
	Retired	224	21.4
	Unemployed	235	24.7

*N=1,051*.

The full survey process was conducted online. A total of 1,051 participants volunteered to answer an online survey. Participants were emailed an anonymous link. Upon clicking the link, they were redirected to a webpage presenting the purpose of the study, which was to investigate relationships among mattering, fairness, and well-being. Consenting was incorporated into the online survey process, and participants who declined to participate, those who were under the age of 18, or who did not reside in the United States were redirected to a thank you page and excluded from the study. Upon completing the survey, eligible participants received a small renumeration for their participation from the survey provider.

#### Demographics

Participants were presented with seven demographic items prior to answering other questions. These included age, marital status, race/ethnicity, gender, annual household income, occupational status, and educational attainment level. Each item was presented as a multiple-choice selection. Quotas were employed to ensure our sample was representative of the U.S. adult population distribution for each variable. Participant demographics are outlined in Table 1, below.

### Measures

A battery consisting of a consent form, a demographics questionnaire, and three main questionnaires, detailed below, was presented to all participants who met the inclusion criteria.

#### Well-being

Well-being was measured using the I COPPE scale short form (Esposito et al., 2021). The I COPPE scale was chosen because of its focus on individual subjective well-being across multiple life domains (Prilleltensky et al., 2015). The short version of this scale uses a Cantril response scale with 14 items. These items provide indicators of present and future subjective well-being in overall well-being and each of the following six domains: Interpersonal, Community, Occupational, Physical, Psychological, and Economic well-being. An example question, addressing Community Well-being in the present, reads as follows:

*This set of questions pertains to your community. The top number ten represents the best your life can be. The bottom number zero represents the worst your life can be. When it comes to the community where you live, on which number do you stand now?*

The I COPPE scale has been validated in several studies with consistently strong psychometric properties in both U.S. and international samples (e.g., Myers et al., 2016; Di Martino et al., 2018; Matera et al., 2020). Moreover, those studies have confirmed that the scale consists of 7 correlated factors. In the present analysis, the I COPPE scale short form showed excellent indices of model fit: $\chi_{\left(42\right)}^{2}$ =70.193, *p*<0.001, CFI=0.99, TLI=0.98, RMSEA=0.03, 90% CI [0.02, 0.04], SRMR=0.01, high composite reliability ranging from a minimum of 0.84 for physical well-being to a maximum of 0.88 for occupational well-being, and high validity, with Average Variance Extracted (AVE) ranging from a minimum of 0.69 for economic well-being and a Maximum of 0.81 for occupational well-being.

#### Fairness

Fairness was assessed using the Multidimensional Fairness Scale (MFS). The MFS consists of 12 items representing four domains of life: interpersonal, occupational, community and society. Hence, it is aligned with the I COPPE scale in measuring fairness at the experiential level across multiple domains of life. Each item features a 5-point Likert scale with the following options: never, rarely, sometimes, often, and always. An example question, which assess community fairness, reads:

*When it comes to your experiences in your local community, how often do you feel that you have the same amount of privileges as everyone else?*

In the present analysis, MFS responses were confirmed as a bifactorial measure of overall fairness as a general factor with domains of life as specific factors. This approach aligns with the theory behind the scale’s construction and validation (Duff, 2016).

In the present sample, the MFS showed acceptable indices of model fit: $\chi_{\left(24\right)}^{2}$ =172.022, *p*<0.001, CFI=0.97, TLI=0.95, RMSEA=0.05, 90% CI [0.04, 0.06], SRMR=0.03. However, the inspection of modification indices and residuals revealed a large unspecified cross-loading between the Societal fairness specific domain and the item “*When it comes to your experiences in your local community … you have the same amount of privileges as everyone else*.” In addition, without this cross-loading, the module could converge only after starting values were significantly increased. Therefore, a decision was made to respecify the model. Although the effect of this item amounted to a relatively small loading onto the Societal fairness domain (*β*=0.35), its presence made the model converge without increasing starting values, and it also greatly improved its overall fit, $\chi_{\left(14\right)}^{2}$ =101.324, *p*<0.001, CFI=0.98, TLI=0.97, RMSEA=0.03, 90% CI [0.02, 0.04], SRMR=0.02. In this model, MFS showed high reliability with omega coefficients (ω) of 0.93 for the general MFS score and values ranging from a minimum of 0.85 for the societal fairness specific domain to a maximum of 0.86 for both interpersonal and occupational fairness specific domains. Lastly, the general fairness domain showed an Explained Common Variance (ECV) of 0.60.

#### Mattering

Mattering was assessed *via* the Mattering in Domains of Life Scale (MIDLS). MIDLS features 27 items and uses a 0–10 Cantril scale and has previously been validated in a U.S. sample (Scarpa et al., 2021b). Like the other two scales employed, MIDLS focuses on individual experiences of mattering across several domains of life (i.e., personal, interpersonal, occupational, and community), each of them representing *feeling valued* and *adding value*, for a total of 8 factors. Each factor is measured through three items which assess one’s level of past, present, the future mattering. Three additional items measure overall mattering in the past, present, and future. The inclusion of past, present, and future items ensures there is more than one item for each subdomain to increase reliability. This approach has been used successfully with other scales which employ a Cantril-type response (e.g., Gallup, 2021; Prilleltensky et al., 2015).

Validation results suggest acceptable psychometric properties and support the suitability of the scale as a bifactorial measure, which build on a general mattering factor and 9 domain-specific subfactors (4 domains for feeling valued, 4 domains for adding value, and 1 overall mattering domain). An example question, which measures Community – Adding Value in the present, reads:

*This set of questions pertains to adding value to your community. This means making a contribution or improving your neighborhood, city, or region in some way. When it comes to adding value to your community, on which number do you stand now?*

In the present analysis, the MIDLS showed acceptable indices of model fit, except for CFI and TLI that were slightly below the recommended thresholds, $\chi_{\left(923\right)}^{2}$ =1015.061, *p*<0.001, CFI=0.94, TLI=0.92, RMSEA=0.05, 90% CI [0.052, 0.059], SRMR=0.06. However, an inspection of modification indices and residuals revealed two large unspecified correlations between the “community feeling valued” and “community adding value” specific domains (*r*=0.73) as well as between the “self adding value” and “overall mattering” specific domains (*r*=0.59). Therefore, the model was respecified to allow those errors to correlate, a condition which considerably increased the fit of the final model, $\chi_{\left(1138\right)}^{2}$ =507.847, *p*<0.001, CFI=0.98, TLI=0.97, RMSEA=0.03, 90% CI [0.02, 0.03], SRMR=0.04. In this final model, the general domain of MIDLS showed high reliability with a value of *ω*=0.98. Additional omega values for the specific domains range from a minimum of 0.88 for the “interpersonal adding value” specific domain and a maximum of 0.94 for “self adding value.”. Lastly, the general mattering factor showed an Explained Common Variance (ECV) of 0.61.

### Analysis

Preliminary analyses and data cleaning were conducted in SPSS version 26.0 (IBM Corp, 2019). Respondents who incorrectly answered a quality check item were removed from the sample. Internal consistency of scales was then calculated using omega coefficients, chosen for their suitability for interpreting a single common factor from multidimensional measures of latent variables (McDonald, 1999; Hancock and An, 2020).

Finally, the hypothesized mediation model was tested through a series of confirmatory factor analyses (CFA), which were used to build a Latent Path Analysis within the framework of structural equation modeling (SEM; Gunzler et al., 2013) with the support of Mplus v. 8 (Muthén and Muthén, 2013). SEM was chosen because of its better correction for measurement error in the use of multi-indicator latent variables when compared to regression-based approaches (Pek and Hoyle, 2016; Wang and Wang, 2019).

Given the presence of multivariate non-normality, maximum likelihood robust (MLR) was used as main estimation in the first model, whereas maximum likelihood with 1,000-sample bootstrapping procedure was employed to calculate standard errors and statistical significance of indirect effects in the second model. To assess the fit of our models, we relied on the cutoff points suggested by Hu and Bentler (1999): chi-square (χ^2^) non-significant at the 5% alpha level, comparative fit index (CFI) and Tucker-Lewis Index (TLI)>0.95, root mean square error of approximation (RSMEA)<0.05, and standardized root mean square residual (SRMR)<0.08. However, we should be mindful that the well-known sensitivity of chi-square test to increasing sample size resulted in statistical significance, due to the relatively large sample we employed in our analyses. Moreover, it is also acknowledged that TLI tends to penalize complex models (Marsh et al., 2004). Given the very large number of parameters in our analyses, we decided to accept values of TLI that were slightly below the recommended threshold, given that all other indices supported the fit of our models.

Missing data were treated with listwise deletion in all cases. This resulted in a minimal loss of 9 cases (0.8% of the total sample) in the final tested SEM model (see Model 2 in the next pages). RMSEA-based power analyses (MacCallum et al., 1996) showed that with 1,142 degrees of freedom and a sample of 1,036 observations, the final SEM model reached a power of 1, and therefore, we can be confident that our results did not incur a type II error.

## Results

To present our results, we follow Kline’s (2016), recommendations to start with the simplest models before testing increasingly complex ones. This is a useful practice to identify any possible misspecification that could otherwise be harder to detect in more complex models. Therefore, we first tested the three main instruments (i.e., I COPPE, MIDLS, and MFS) separately. The main results have been presented above and can be found in each measure’s respective sub-paragraph. Having assessed the psychometric characteristics of the instruments under examination, we included them together in a latent path analysis in which the 7 domains of well-being (I COPPE) were regressed onto the general Mattering (MIDLS) and Fairness (MFS) factors. Additionally, the general mattering factor was regressed onto the general fairness factor. Given the presence of two bi-factor structures, we set to zero all correlations between the specific domains of both MIDLS and MFS and their respective general domains. All the error terms between the specific domains of MIDLS and MFS were left free to correlate, except for non-significant paths, which were set to zero to save on degrees of freedom.

The hypothesized Model 1 showed adequate fit, $\chi_{\left(1138\right)}^{2}$ =2669.374, *p*<0.001, CFI=0.95, TLI=0.94, RMSEA=0.03, 90% CI [0.034, 0.038], SRMR=0.05. In terms of measurement model, the three scales put together present high and significant standardized structural coefficients, which indicate adequate construct validity and reliability, with values very similar to those obtained when they were examined separately (see Table 2). In addition, discriminant validity is supported by intercorrelations between latent constructs never exceeding 0.9 (see Kline, 2016), with the highest value found between Overall mattering and Overall well-being (*r*=0.84).

**Table 2:** Validity and reliability measures of the Measurement model.

**I COPPE scale short form**		
		Standardized structural coefficients^*^	Composite reliability	AVE			
Overall well-being	Present	0.92	0.85	0.74			
	Past	0.80					
Interpersonal well-being	Present	0.93	0.87	0.77			
	Past	0.83					
Community well-being	Present	0.92	0.88	0.79			
	Past	0.86					
Occupational well-being	Present	0.93	0.81	0.89			
	Past	0.88					
Physical well-being	Present	0.94	0.74	0.85			
	Past	0.79					
Psychological well-being	Present	0.91	0.77	0.87			
	Past	0.85					
Economic well-being	Present	0.87	0.69	0.82			
	Past	0.80					
**Mattering in Domains of Life Scale (MIDLS)**				
		**Specific factors**			**General factors**		
		**Standardized structural coefficients**	**OmegaS**	**ECV_S**	**Standardized structural coefficients**	**Omega**	**ECV**
Self (feeling valued)	Present	0.57	0.89	0.3	0.76	0.98	0.61
	Past	0.42			0.65		
	Future	0.43			0.74		
Interpersonal (feeling valued)	Present	0.58	0.88	0.27	0.76		
	Past	0.39			0.67		
	Future	0.35			0.74		
Occupational (feeling valued)	Present	0.49	0.91	0.26	0.78		
	Past	0.44			0.72		
	Future	0.41			0.77		
Community (feeling valued)	Present	0.73	0.92	0.62	0.57		
	Past	0.67			0.53		
	Future	0.72			0.55		
Self (adding value)	Present	0.62	0.94	0.39	0.73		
	Past	0.57			0.71		
	Future	0.54			0.70		
Interpersonal (adding value)	Present	0.47	0.88	0.21	0.80		
	Past	0.32			0.68		
	Future	0.38			0.76		
Occupational (adding value)	Present	0.57	0.9	0.34	0.73		
	Past	0.52			0.65		
	Future	0.44			0.72		
Community (adding value)	Present	0.68	0.92	0.56	0.63		
	Past	0.65			0.55		
	Future	0.69			0.61		
Overall mattering	Present	0.60	0.92	0.41	0.71		
	Past	0.57			0.67		
	Future	0.55			0.68		
**Multidimensional Fairness Scale (MFS)**				
Interpersonal fairness	MFS_I_1	0.50	0.86	0.42	0.60	0.93	0.6
	MFS_I_2	0.59			0.64		
	MFS_I_3	0.52			0.64		
Occupational	MFS_O_4	0.56	0.86	0.61	0.54		
	MFS_O_5	0.72			0.54		
	MFS_O_6	0.65			0.46		
Community	MFS_O_7	0.24	0.73	0.15	0.60		
	MFS_O_8	0.37			0.64		
	MFS_O_9	0.19			0.67		
Societal	MFS_O_10	0.41	0.78	0.35	0.70		
	MFS_O_11	0.66			0.65		
	MFS_O_12	0.20			0.68		
MFS_O_8	0.38			

* *All coefficients are statistically significant at the 1% alpha level*.

*N.B. Ave*, *Average Variance Extracted; OmegaS, Omega coefficient for specific factors; Omega*, *Omega coefficient for general factors; ECV_S, Explained common variance for specific factors; ECV*, *Explained common variance for general factors*.

Turning to the structural model, we notice that the general domain of the MIDLS significantly predicts all of the 7 I COPPE domains of well-being with high standardized regression coefficients (see Table 3), ranging from a minimum effect on Economic Well-being, *β*=0.54, *p*<0.001, 95% CI [0.46, 0.63], *R*^2^=0.59, to a maximum effect on Overall well-being, *β*=0.79, *p*<0.001, 95% CI [0.73, 0.86], *R*^2^=0.71. In turn, the MFS general domain significantly and highly predicts the general domain of MIDLS, *β*=0.67, *p*<0.001, 95% CI [0.62, 0.72], *R*^2^=0.45.

**Table 3:** Standardized Coefficients for Model 1 and Model 2.

	Model 1			Model 2		
	*β*	*p*	CI	*β*	*p*	CI
**Direct effects**
**Mattering predicting well-being**						
MIDLS → OWB	0.79	<0.001	0.73, 0.86	0.84	<0.001	0.80, 0.87
MIDLS → IWB	0.73	<0.001	0.66, 0.81	0.79	<0.001	0.75, 0.82
MIDLS → CWB	0.70	<0.001	0.62, 0.77	0.73	<0.001	0.66, 0.78
MIDLS → OWB	0.65	<0.001	0.56, 0.74	0.70	<0.001	0.65, 0.75
MIDLS → PHWB	0.71	<0.001	0.62, 0.79	0.74	<0.001	0.68, 0.78
MIDLS → PSWB	0.74	<0.001	0.66, 0.81	0.76	<0.001	0.70, 0.82
MIDLS → EWB	0.54	<0.001	0.46, 0.63	0.57	<0.001	0.50, 0.64
**Fairness predicting mattering**						
MFS → MIDLS	0.67	<0.001	0.62, 0.72	0.68	<0.001	0.64, 0.72
**Fairness predicting well-being**						
MFS → OVWB	0.06	0.113	−0.01, 0.14	NA	NA	NA
MFS → IWB	0.08	0.06	−0.0, 0.16	NA	NA	NA
MFS → CWB	0.13	0.001	0.05, 0.21	0.09	0.01	0.02, 0.16
MFS → OWB	0.06	0.189	0.03, 0.16	NA	NA	NA
MFS → PHWB	0.04	0.391	−0.05, 0.13	NA	NA	NA
MFS → PSWB	0.11	0.005	0.03, 0.19	0.07	0.03	0.004, 0.13
MFS → EWB	0.28	<0.001	0.20, 0.37	0.24	<0.001	0.16, 0.31
**Total Indirect effects**							**Mattering mediating through fairness and well-being**						
	*β*	*p*	**BS CI**	*β*	*p*	**BS CI**
MFS → MIDLS → OVWB	0.54	<0.001	0.47, 0.60	0.58	<0.001	0.53, 0.62
MFS → MIDLS → IWB	0.49	<0.001	0.43, 56	0.54	<0.001	0.49, 0.59
MFS → MIDLS → CWB	0.47	<0.001	0.41, 0.54	0.50	<0.001	0.44, 0.55
MFS → MIDLS → OWB	0.44	<0.001	0.37, 0.51	0.48	<0.001	0.43, 0.53
MFS → MIDLS → PHWB	0.48	<0.001	0.41, 0.55	0.50	<0.001	0.46, 0.55
MFS → MIDLS → PSWB	0.50	<0.001	0.43, 0.56	0.52	<0.001	0.47, 0.58
MFS → MIDLS → EWB	0.37	<0.001	0.30, 0.43	0.39	<0.001	0.34, 0.44
**Total direct and indirect effects combined**						
MFS → MIDLS → CWB+MFS → CWB	0.60	<0.001	0.55, 0.66	0.59	<0.001	0.54, 0.64
MFS → MIDLS → PSWB + MFS → PSWB	0.61	<0.001	0.55, 0.66	0.60	<0.001	0.54, 0.64
MFS → MIDLS → EWB+MFS → EWB	0.65	<0.001	0.60, 0.71	0.64	<0.001	0.58, 0.69

*β=Standardized estimates, p=statistical significance (p value), CI=95% Confidence Intervals, BS CI=95% 1,000 Bootstrapped Confidence Intervals, MIDLS=Mattering in Domains of Life Scale, MFS=Multidimensional Fairness Scale, OVWB-EWB=Overall, Interpersonal, Occupational, Physical, Psychological and Economic well-being*.

However, the model also shows that only three out of the seven I COPPE domains are significantly associated with the general domain of MFS. Among these, the strongest direct effect was found on Economic well-being, *β*=0.28, *p*<0.001, 95% CI [0.20, 0.37], followed by Community well-being, *β*=0.13, *p*=0.001, 95% CI [0.05, 0.21], and Psychological well-being, *β*=0.11, *p*=0.005, 95% CI [0.03, 0.19].

Based on these findings, we tested a new model (Model 2) including only significant effects from the general domain of MFS on I COPPE. In other words, this model tested the hypothesis of mattering fully mediating the relationship between fairness and overall, interpersonal, occupational, and physical well-being while partially mediating the relationship between fairness and community, psychological, and economic well-being. To test for indirect effects, we relied on maximum likelihood estimator with Bootstrapped standard error and 95% confidence intervals. Compared to the previous model, Model 2 shows slightly poorer fit – a condition mainly due to the use of ML rather than MLR estimator in the presence of multivariate non normal distributed data variables – although the indices are still within acceptable range, $\chi_{\left(1142\right)}^{2}$ =4007.069, *p*<0.001, CFI=0.94, TLI=0.93, RMSEA=0.04, 90% CI [0.048, 0.051], SRMR=0.05.

**Figure 1:**
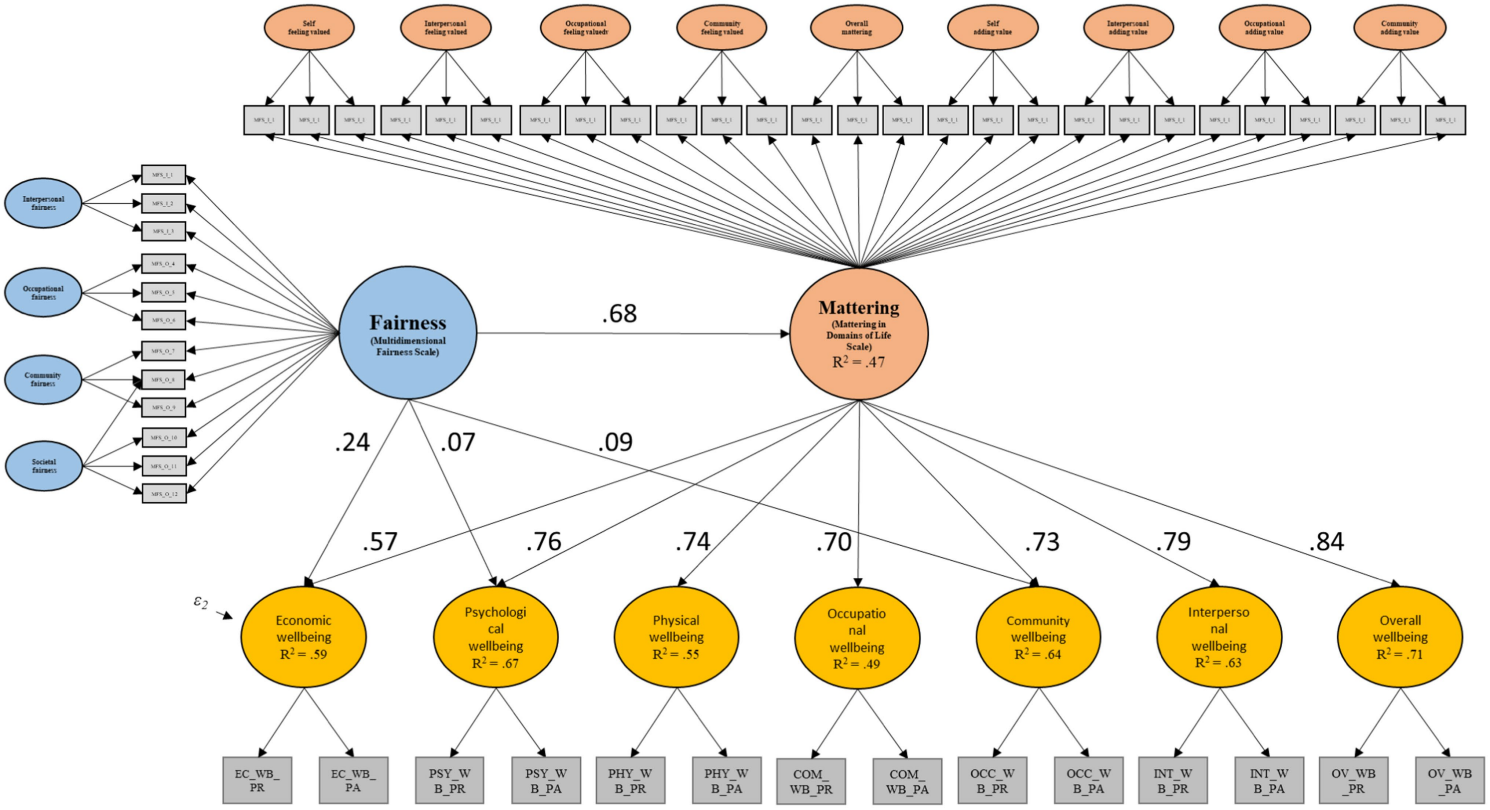
Main effects of Model 2 with standardized coefficients. N. B. All displayed results are significant at the 0.1% alpha level. Only significant standardized regression coefficients between latent variables are reported to reduce clutter.

Model 2 presents similar results to Model 1 in terms of direct effects. Figure 1 shows the main results of Model 2 in a graphical format.

As we can see from the figure above, once more the general domain of MIDLS is significantly related to all the seven domains of I COPPE. Additionally, the MFS significantly predicts the I COPPE domains of Community, *β*=0.13, *p*=0.001, 95% CI [0.05, 0.21], Psychological, *β*=0.11, *p*=0.005, 95% CI [0.03, 0.19], and Economic well-being, *β*=0.37, *p*<0.001, 95% CI [0.30, 0.43]. However, it is worth reporting that, except for the latter case, the first two coefficients present an extremely small effect. In addition, *p* value and confidence intervals are on the verge statistical significance; therefore, the hypothesis of full mediation should not be discarded.

In terms of indirect effects, strong and significant paths were found stemming from the general domain of MFS onto all the seven I COPPE domains of well-being through the general domain of MIDLS (see Table 3). Among the fully mediated paths, the largest indirect effect was found on overall well-being, *β*=0.58, *p*<0.001, 95% CI [0.53, 0.62], with *R*^2^ accounting for 71% of the total variance, and the smallest on occupational well-being, *β*=0.48, *p*<0.001<0.001, 95% CI [0.43, 0.53], with *R*^2^ accounting for 49% of the total variance. Among the total indirect effects – which account for the additional direct effect of the general domain of MFS onto the I COPPE domains – the path stemming from MFS through MIDLS onto economic well-being, with the additional path of MFS onto economic well-being, shows the strongest significant total effect, *β*=0.64, *p*<0.001, 95% CI [0.58, 0.69], with *R*^2^ accounting for 59% of the total variance, whereas the smallest effect was found on community well-being, *β*=0.59, *p*<0.001, 95% CI [0.54, 0.64], with *R*^2^ accounting for 64% of the total variance. For parsimony, these calculated values are not displayed in Figure 1.

## Discussion

The results support the hypothesis that mattering mediates the relationship between fairness and well-being. In fact, our findings showed that the seven domains of well-being measured by the I COPPE scale short form are all significantly and strongly associated with mattering, as measured by the Mattering in Domains of Life Scale. In turn, the latter is strongly and significantly linked to fairness as measured by the general domain of the MFS. The results also show that only in the case of economic well-being, there is clear evidence of partial mediation. For all the other domains of well-being, the evidence points toward mattering fully mediating the relationship between fairness and well-being. This also potentially applies to community and psychological well-being, whose relationship with fairness is supported by such small and close to non-statistical significance that the hypothesis of full mediation is more plausible than the one of partial mediation.

The full mediation relationship reported here points to mattering as an important mechanism that can explain how fairness impacts on wellness. The divergent finding of a partial mediation relationship between fairness and economic well-being suggests that, in the presence of fairness, mattering is not the only element directly predicting economic well-being. Further, our findings build upon established literature connecting both fairness and mattering to well-being, thereby offering novel evidence concerning the relationship between these two constructs. While prior studies have started to explore the relationship between mattering and fairness (e.g., Lachance-Grzela, 2012), to our knowledge, this is the first empirical large-scale investigation that provides evidence of the predictive power of fairness onto mattering.

### Theoretical Implications

This study offers several implications for theory, research, and practice. At the theoretical level, it shows that fairness exerts an impact on wellness mainly through experiences of mattering, which, as noted earlier, consist of feeling valued and adding value. This points toward a distinct human element to fairness, since we can assume that the more we experience fairness in relationships, at work, and in society at large, the more likely we are to also feel that our life matters. Likewise, when people and institutions treat people with dignity and respect and accord them their fair due, they are more likely to feel that they matter as human beings. Although there are many studies, summarized above, demonstrating the connection between fairness and wellness, this study offers a clear demonstration that the effect is mediated through feelings of mattering.

Regarding research, it is important to explore possible relationships among fairness, mattering, and well-being at more granular levels of analysis. The scales used in this study are all multidimensional, and future investigations can ascertain if more fairness at work predicts more mattering at work and if both predict occupational well-being. Our mediation model used primarily the total score of the fairness and mattering scales to predict various domains of well-being, but subscale scores could also be used to understand more contextually how fairness impacts on specific areas of both mattering and wellness. Further implications for researchers are explored below.

### Practical Implications

There are also practical implications for professionals, policy makers, and agents of social change. Professionals in education, psychology, social work, economics, medicine, and counseling must pay attention to the importance of individuals feeling like they matter when interacting with experts (Prilleltensky and Prilleltensky, 2021). Potentially, a person can live in a community where there is a good measure of distributive justice, but if government personnel is indifferent to people and community members are treated like numbers, the salutary effect of socially just policies is significantly diminished. The opposite can also be true. Practitioners may be exceedingly caring and sensitive to the plight of minorities, but if the government fails to provide basic necessities, all the humane caring in the world will not provide shelter, food, and education for refugees. For communities to thrive, we need both objective resources and subjective caring and compassion. Practitioners should also keep in mind that it is not enough to make people feel valued. They also need to create opportunities for citizens to add value through work, study, or volunteer opportunities.

When it comes to agents of social change, the present study suggests that we should never treat people as means to an end. If we want to create a society where everyone matters, we must practice compassion and caring with our peers and allies. There have been documented cases where social justice movements have sacrificed relational welfare for the ultimate cause of justice. Such approaches risk activists feeling like they do not matter and dropping out (Prilleltensky and Prilleltensky, 2021).

At the policy level, there is evidence that fairness leads to higher levels of satisfaction for entire populations (Di Martino and Prilleltensky, 2020). To create optimal conditions for the common good, objective conditions of fairness must improve, and subjective experiences of mattering must be promoted by professionals, experts, and citizens alike. It is when both objective and subjective needs are satisfied that populations thrive. Flett and Zangeneh (2020) illustrate this notion through a focus on responses to COVID-19. In addition to providing medical attention and vaccination, they argue, communities and governments should attend to the psychological and relational mattering needs that have gone unmet during the pandemic. This is especially important for vulnerable and marginalized populations who face the greatest stress during periods of difficulty and loss.

### Limitations and Future Considerations

Several limitations deserve acknowledgment. Since this is a cross-sectional study, our findings cannot demonstrate a causal relationship between the variables employed in our model. Relatedly, Little et al. (2007) articulate two relevant interpretation issues for mediation models. First, while our results provide support for the hypothesized mediation relationship, they do not rule out alternative possible models of the relationship between our variables. Second, given the conceptual breadth of mattering, well-being, and fairness, unmodeled correlates related to the constructs investigated in our study may have great explanatory importance. For instance, while several demographic variables were collected for the purpose of ensuring sample quality, none were used as controlling, moderating, or mediating variables in our model. This treatment aligned with our hypothesis-driven investigation into the overall relationships between these constructs at the population level, but possible interactions between demographic factors and our mediation relationship present important questions.

Next, this study used a single, large, U.S., English-speaking sample. While the theoretical rationale underlying our hypotheses takes all three constructs to be fundamental human needs, the shape and nature of their connections, and the conditions under which they are satisfied, are likely to be mediated by culture (de Oliveira, 2013). It may be the case that, as has been found for the relationship between justice and well-being (Di Martino and Prilleltensky, 2020), the mediation model reported here fits across cultures but to varying degrees or with path alterations. Such an outcome would also resemble findings concerning the similar relation between self-determination and well-being in Bulgaria and the United States (Deci et al., 2001). Another possibility is that mattering, fairness, and well-being share cross-cultural relevance, but that cultural factors influence the directionality of mediation. Such a difference has been demonstrated regarding the relationships between friendship, mattering, and happiness in the United States and Turkey (Demir et al., 2012). Ultimately, our data do not allow us to distinguish between these and related possibilities. Additional studies in international and non-English-speaking contexts are necessary to determine the cross-cultural salience, applicability, and determinants of the relationship between mattering, fairness, and well-being.

Further, results that hold over a general population may not be true for particular groups within that population (Rowe and Trickett, 2018; Buchanan et al., 2020). This is a meaningful concern given the demographic heterogeneity of the United States and prior findings that mattering levels vary within-country race/ethnicity, gender, age, and other demographic groups (Scarpa et al., 2021a). As such, focused studies exploring the experiences of marginalized and underrepresented groups should be undertaken. This is doubly important in light of the importance of mattering to social justice movements, such as *Black Lives Matter*, the LGBTQ+ community, and the struggle for decolonization and indigenous rights.

Limitations notwithstanding, the multidimensional approach used in the present analysis suggests compelling directions for further study. One possible direction involves connecting mattering, fairness, and well-being to the concepts of participation and citizenship. Several authors have bemoaned the absence of psychologists from discussions of citizenship, as psychology may be uniquely suited to explore the needs and tendencies of democratic subjects in social contexts (Condor, 2011; Andreouli, 2019). Mattering, which has a growing psychological tradition, represents a promising linkage between psychology and the study of citizenship. In particular, participation can be understood as both a key component of citizenship (von Heimburg et al., 2021) and as a means of mattering by *adding value*.

## Conclusion

Though prior literature has suggested that fairness is indispensable to human well-being, relatively little psychological research has illuminated specific pathways through which this is the case. Mattering – the experience that one feels valued and can add value to various domains of life – is one such potential mechanism. The present results contribute to the literature by providing evidence that mattering mediates between fairness and well-being in a representative U.S sample. Future studies should investigate the extent to which this mediation relationship exists in other linguistic and cultural contexts, and what cultural factors may influence it.

Our findings have implications for the concepts of citizenship and participation. Although we did not empirically test the relationship between our constructs and citizenship, our findings suggest theoretical implications of how the relationship between mattering, fairness, and well-being can also shape people’s experience of citizenship. Citizenship is comprised both of conventional and transformative aspects; citizens “do not just obey the rules; they can, and do, contest them” (Andreouli, 2019 p. 3). In addition to sense of belonging, citizenship is about the struggle to change and improve communities. In such transformative engagement, we believe, mattering is realized.

## Data Availability Statement

The raw data supporting the conclusions of this article will be made available by the authors, without undue reservation.

## Ethics Statement

The studies involving human participants were reviewed and approved by University of Miami Institutional Review Board. The patients/participants provided their written informed consent to participate in this study.

## Author Contributions

MS led data collection as well as the authorship of the article and wrote most sections. SM led the analysis and authored the analysis and results suggestions as well as related tables and figures, and contributed throughout. IP conceived the hypothesis and contributed to several sections, including substantial sections of the discussion. All authors contributed to the article and approved the submitted version.

## Funding

This project was funded by the Erwin and Barbara Mautner Endowed Chair in Community Well-Being at the University of Miami. The third author of this paper is the holder of the Endowed Chair.

## Conflict of Interest

The authors declare that the research was conducted in the absence of any commercial or financial relationships that could be construed as a potential conflict of interest.

## Publisher’s Note

All claims expressed in this article are solely those of the authors and do not necessarily represent those of their affiliated organizations, or those of the publisher, the editors and the reviewers. Any product that may be evaluated in this article, or claim that may be made by its manufacturer, is not guaranteed or endorsed by the publisher.
